# Inverted Molecular Beacons as Reaction-Based Hybridization Probes for Small-Molecule Activation by Nucleic Acid Inputs

**DOI:** 10.1021/acschembio.5c00333

**Published:** 2025-07-15

**Authors:** Cole Emanuelson, Anirban Bardhan, Nicholas Ankenbruck, Jessica Boette, Alexander Deiters

**Affiliations:** Department of Chemistry, 6614 University of Pittsburgh, Pittsburgh, Pennsylvania 15260, United States

## Abstract

Nucleic acid–based hybridization probes that produce a fluorescent signal in the presence of DNA or RNA target molecules are essential components of nucleic acid computation and detection strategies. Commonly, the fluorescence activation of reporter gates is triggered by separation of a fluorophore–quencher pair upon target hybridization or strand displacement. In order to expand the utility of DNA computing by providing a chemical reaction as the ultimate output, reporter systems have been designed that carry reactive groups, which undergo a proximity-induced reaction upon oligonucleotide hybridization. The downside of published reporter gate designs is that they are composed of two separate, chemically modified oligonucleotides, which need to be taken into consideration when designing upstream circuits. Here, we report a novel hairpin-forming nucleic acid reporter probe that utilizes template-induced proximal reactivity to activate a small molecule in the presence of an unmodified nucleic acid input molecule. This DNA hairpin reporter gate consists of a duplex between a blocking strand and a hairpin-forming reporter strand. In the presence of input, the blocking strand is displaced, triggering hairpin formation allowing the proximity-driven templated activation of a vinyl ether-caged fluorophore by a tetrazine via an inverse electron demand Diels–Alder reaction. This new approach demonstrates robust small-molecule activation *in vitro* and in cells through logic operations in the presence of input DNA molecules.

## Graphical abstract

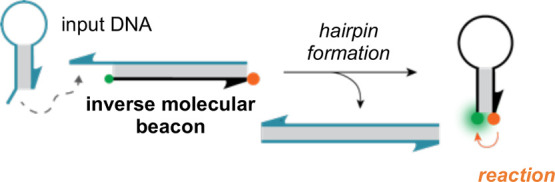

## Introduction

Probes that report on the detection of nucleic acids through the production of a fluorescent signal are important tools for studying biological systems and as molecular diagnostics.^1,2^ Many probe detection systems traditionally rely on FRET-type fluorescence quenching to regulate the probe activation. Most of these quencher-based probes can be categorized into two classes: (a) a double-stranded oligonucleotide consisting of a fluorophore–quencher pair at one end^3–5^ and (b) a molecular beacon, consisting of a stem (5–7 nt) and a variable loop region (18–30 nt) along with a fluorophore–quencher pair on the termini.^6–8^ Molecular beacons are oligonucleotide probes that allow targeted DNA and RNA detection and quantification,^8–12^ as well as small molecule and protein sensing.^13^ The stem loop or the hairpin motif is critical to the functioning of a molecular beacon. Almost all these applications involve the activation of the molecular beacon from the inactive/closed state where the proximity of the fluorophore and quencher blocks the signal to the active/open state, in which upon binding of the molecular beacon to its target, the fluorophore and the quencher are separated and the beacon becomes fluorescent (Figure 1).

**Figure 1:**
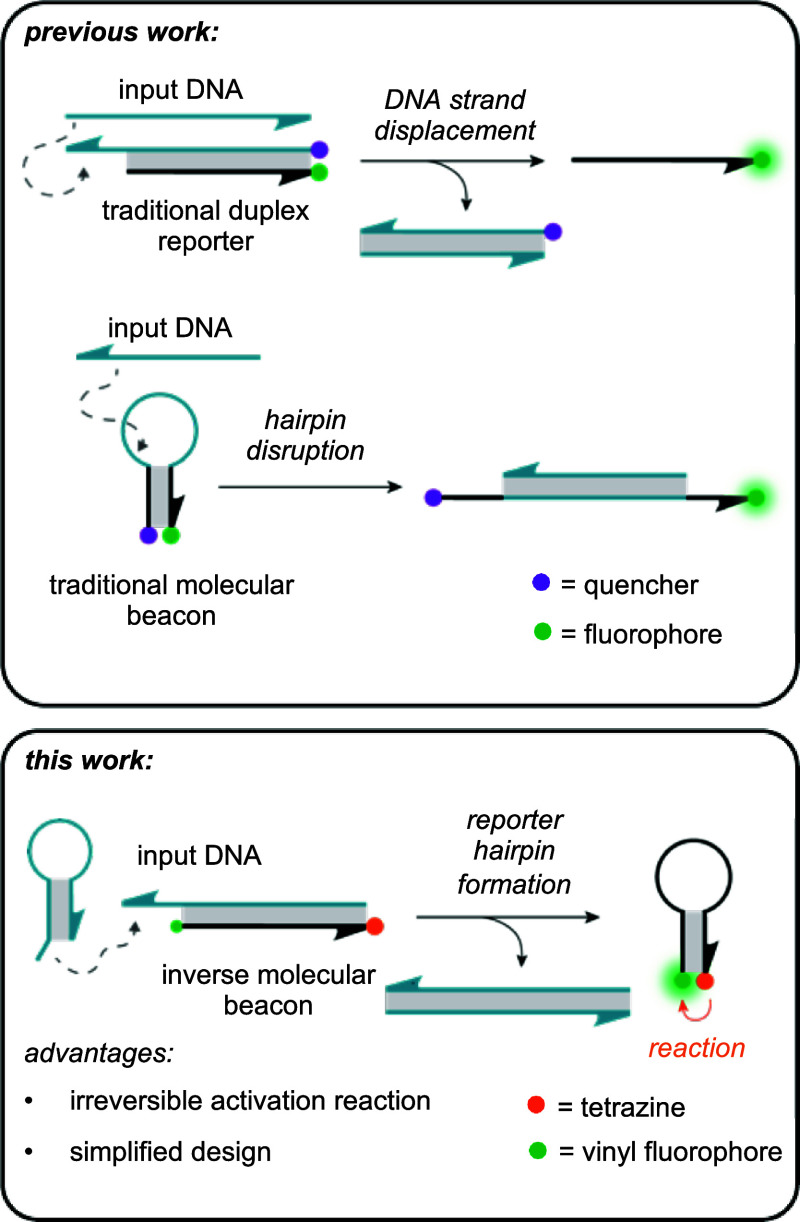
Previous work detecting nucleic acids routinely utilizes traditional FRET-based fluorophore–quencher probe pairing to achieve activation. Examples include strand displacement schemes for a duplex reporter and a molecular beacon, wherein an input DNA facilitates the separation of a fluorophore and a quencher pair. In this work, an inverse molecular beacon-based reporter is introduced, which is activated by input DNA-initiated strand displacement, hairpin formation, and a proximity-induced templated inverse electron demand Diels–Alder reaction.

DNA computation is based on Boolean logic operations (AND, OR, and NOT) implemented as a series of toehold-mediated strand displacement reactions between input oligonucleotides and semistable multistrand gate complexes.^14,15^ They can be assembled into complex DNA circuits that can detect a variety of input patterns and carry out sophisticated computations, including neural networks,^16^ pattern generation,^17^ execution of 6-bit algorithms,^18^ and data storage and retrieval.^19^ Such logic operations traditionally use double-stranded DNA modified with a fluorophore–quencher pair as the reporter gate, where a fluorescence output is generated upon displacement of the fluorophore-modified strand, separating it from the quencher (Figure 1).^4,14^ While the use of traditional FRET-based systems—multistrand or molecular beacon reporters—in DNA computation has enabled the implementation of a variety of computation circuits, the unquenching that results from these systems has limited utility aside from the production of a fluorescent signal. To overcome this limitation, we developed probes that undergo a chemical reaction in response to a nucleic acid input. Such probe activation through templated chemistry is capable of enabling the conditional triggering and/or release of molecules in response to a specific input with potential functionality that exceeds a fluorescence quencher displacement.^20^

Herein, we report an approach that utilizes an inverse molecular beacon with chemically modified termini as the chemical-reaction-based reporter gate (Figure 1). Unlike the traditional FRET reporters where the toehold-mediated strand displacement separates the fluorophore-modified strand from the quencher strand, this hairpin design produces an output when the hairpin undergoes folding upon displacement of a blocking strand. The reporter only produces a fluorescent signal when the reactant pair at the now proximal termini undergoes a bioorthogonal reaction. Significantly, the unique inverse nature of this reporter design only requires a single modified oligonucleotide, in contrast to traditional FRET reporters and other examples of DNA-templated activation that rely on the use of multiple chemically modified strands for gate assembly. We chose an inverse electron demand Diels–Alder (IEDDA) reaction as the click reaction of choice, since it is highly efficient in activating and releasing small molecules.^21,22^ Other chemical reactions that have been adapted for DNA-templated chemistry include Staudinger reduction,^23^ thiol–disulfide exchange,^24^ azide–alkyne cycloaddition,^25,26^ and Heck coupling.^25,26^ IEDDA reactions possess favorable characteristics such as fast reaction kinetics and stable reaction partners that make them an ideal choice for use in living cells.^27,28^ The work described here focuses on the development of a DNA hairpin-templated IEDDA reporter that is triggered by the output of a DNA strand displacement cascade. The reactant pair consists of a vinyl-ether-caged fluorescein and a methyltetrazine. These probes are inherently stable molecules under physiological pH and only react when brought in close proximity.^29,30^ Thus, the favorable physiochemical properties of these probes permits the delivery and characterization of DNA-based circuits, utilizing the inverse molecular beacon reporter gate both *in vitro* and within living cells.

## Results and Discussion

The inverse molecular beacon reporter consists of a duplex between two complementary DNA strands: a blocking strand and a dual-modified hairpin-forming strand (Figure 1). We chose a 29 nt hairpin strand that had a 5 bp stem domain and a 19 nt loop domain to achieve high stability of the molecular beacon (**HP1**, Supporting Table S1) following standard design rules.^31^ Other designs matching individual circuit and sequence requirements are certainly conceivable. The GC-rich stem sequence was selected to further provide favorable thermodynamics, while the loop region was designed to facilitate strand displacement of the blocking strand from the molecular beacon. The blocking strand (35 nt, **HPRblock**, Supporting Table S1), which linearizes the reporter strand, has six additional nucleotides that serve as a toehold for a strand displacement reaction in the presence of an input strand. The relative thermodynamic stabilities of the current DNA complexes formed at each step in the desired activation pathway were evaluated using the mFold Web server, which predicted *T*_m_ values of 65, 71, and 78 °C, for the **HP1** hairpin, **HP1**-**HPRblock** duplex, and **HPRblock**-**input_th6** duplex, respectively.^32^ Additionally, the nucleic acid analysis web app NUPACK was used to assess the equilibrium complex concentration of the **HPRblock** and **HP1** strands in the presence and absence of **input_th6**.^33^ This analysis predicted near-complete formation of the **HP1**-**HPRblock** complex in the absence of input DNA and full hairpin, **HP1**, and **HPRblock**-**input_th6** duplex formation in the presence of input DNA, with complexes composed of other combinations of these strands making up less than 0.1% of the concentration of the desired product (Supporting Figure S1). Similar to other toehold-mediated strand displacement designs, the inverse molecular beacon reporter exists as a stable duplex until hybridization of a fully complementary input to the single-stranded toehold of the blocking strand initiates a process of branch migration and eventual displacement of the hairpin-forming strand that is net energetically favorable.^34^

For the assembly of the caged hairpin reporter strand, we utilized a 5′-alkyne and 3′-amine modified DNA strand **HP1**, which enables conjugation of the reactant pairs to the strand. We chose a vinyl ether-caged fluorescein and a tetrazine as the reactive partners (Figure 2A) since they have been utilized to undergo IEDDA reaction under templated conditions.^29^ When the hairpin folds and the reactant pair comes in close proximity, an IEDDA reaction occurs to free the masked hydroxyl group of the fluorescein, thereby restoring fluorescence and activating the reporter. We synthesized the vinyl ether-caged fluorescein (**1**) with an azide handle while the methyltetrazine (**2**) was generated as an active NHS ester (Supporting Scheme S1), adapting the synthesis from a previous report.^35^ A second tetrazine, pyridyltetrazine NHS ester **3**, was generated using a similar protocol. We wanted to evaluate both tetrazine reactant pairs as the pyridinyl tetrazine has reportedly faster kinetics than the methyltetrazine.^22,36^ Following the successful synthesis of the small-molecule compounds, we first treated the 5′-alkyne-3′-amine-modified hairpin strand **HP1** with **1** to undergo a copper-catalyzed azide alkyne cycloaddition (CuAAC) reaction, yielding the 5′-vinyl ether-caged fluorescein-modified intermediate strand **HP2** (Figure 2A), which was characterized by ESI-HRMS (calculated: 9886.68 Da, observed: 9888.90 Da; SMS Spectra—Supporting Information). We observed clean conversion of HP1 to HP2, which is consistent with the reaction of single-stranded amino modified oligonucleotides with a fluorophore NHS ester.^29,30,37^ Because hairpin formation is enthalpically favored and premature formation would cause a reaction between the two reactive groups, a linearized reporter gate duplex was necessary before the installation of the methyltetrazine. Thus, the singly modified **HP2** strand was linearized through duplex formation using a slight excess (1.5 equiv) of the complementary blocking strand (**HPRblock**,Supporting Table S1). With the **HP2** termini separated, the DNA duplex was reacted with an NHS ester-methyltetrazine **2** or -pyridinyl tetrazine **3** to generate the final reporter gates **HP3** or **HP4**, respectively. The conversion to **HP3** or **HP4** was moderate compared to **HP2**, with the difference being modification of double-stranded vs single-stranded DNA. The gates were purified by reverse-phase chromatography and resuspended in TE-Mg^2+^ buffer for storage at 4 °C and subsequent use in downstream assays. The final reporter gates were characterized by ESI-HRMS, where the duplex gates upon ionization led to observation of the masses, indicative of the single-stranded DNAs that constituted each reporter gate (HPR block and HP2-(Me-tetrazine) for the HP3 gate; HPR block and HP2-(Py-tetrazine) for HP4; MS Spectra—Supporting Information). Although we were not able to obtain complete conversion of duplex **HP2**, there was sufficient resolution between unreacted **HP2** and product **HP3** or **HP4** during chromatographic separation to isolate the desired product (Supporting Figure S2).

**Figure 2:**
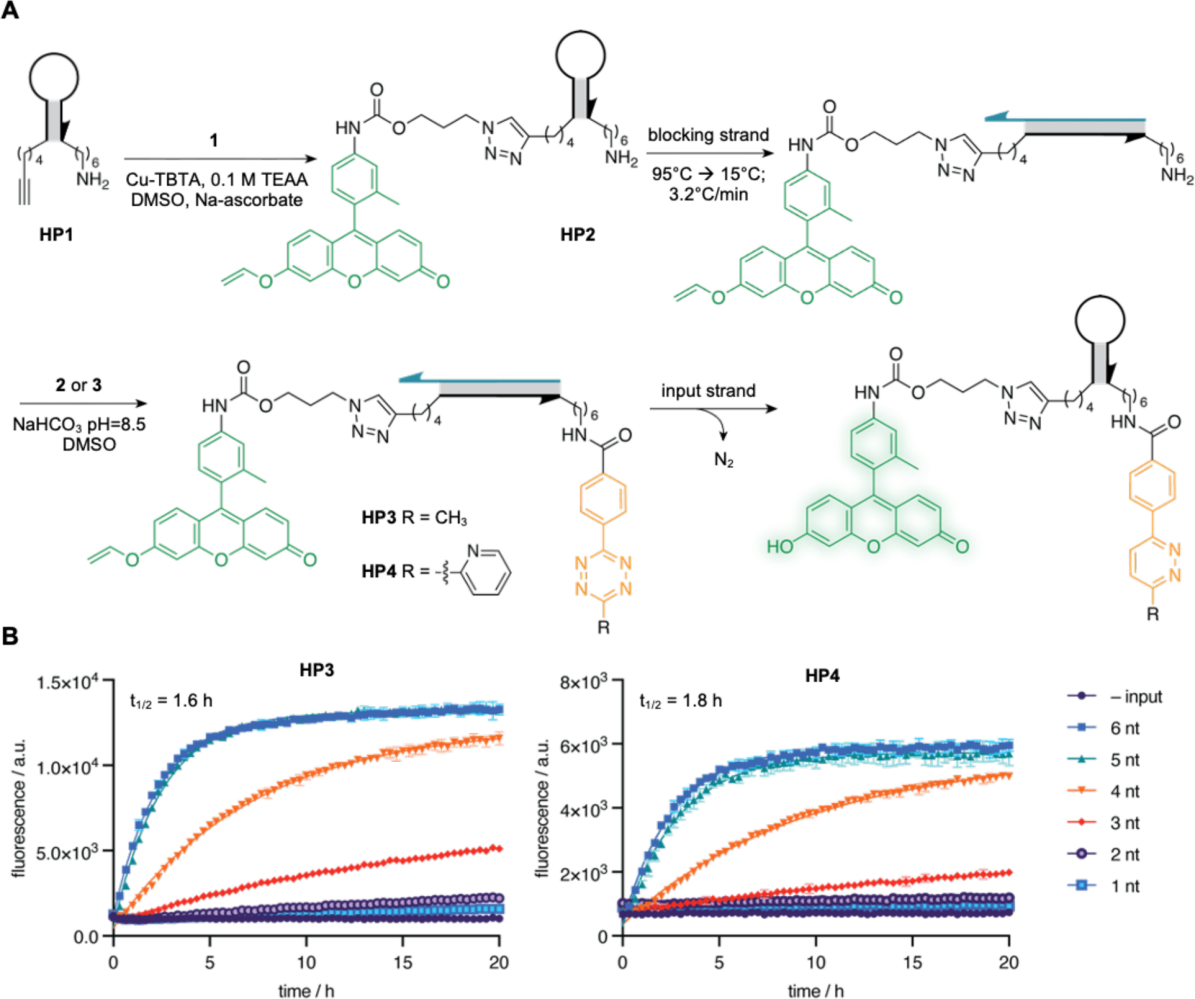
Synthesis and characterization of the hairpin-templated reporter. (A) Schematic showing the generation of final reporter gates **HP3** and **HP4** starting from **HP1** as the starting oligonucleotide and subsequent activation of HP3/HP4 in the presence of an input strand. Reaction of **HP1** (harboring an alkyne at the 5′-end and an amino handle at the 3′-end) with fluorescein azide **1** under CuAAC conditions yields **HP2**, which then undergoes annealing with a complimentary strand to yield the duplex **HP2**. The duplex **HP2** then reacts with tetrazine NHS esters **2** and **3** to generate the final reporter gates **HP3** and **HP4**, respectively. (B) *In vitro* fluorescence time course activation of the caged reporter (50 nM) in the presence and absence of input DNA (1.25×, 62.5 nM) with varying toehold lengths with incubation at 37 °C. Mean fluorescence is shown ± s.d. *n* = 3.

Following successful synthesis, the activities of the inverse molecular beacon reporters **HP3** and **HP4** were evaluated using an *in vitro* fluorescence assay. In our design, there are three distinct steps that take place for the reporter gate activation: toehold-mediated strand displacement of the blocking strand (**HPRblock**) by the input strand, folding of the termini-modified hairpin strand, and IEDDA reaction between the biorthogonal reactant pairs at the hairpin termini. The kinetics of the strand displacement reaction varies over a factor of 10^6^, from *k*_2_ = 1 to 6 × 10^6^ M^–1^ s^–1^, and is mostly dependent on the length and sequence of toehold domain on the invading/migrating strand.^4^ Thus, the length of the toehold domain will greatly affect the kinetics of the overall reporter activation. It is also anticipated that the impact of toehold length will outweigh the contribution of the inherent hairpin structure of the input strand on the kinetics of reporter activation, as has been shown in other investigations into hybridization of complementary hairpin strand possessing a 6 nt external toehold.^38^ Next, the kinetics of hairpin folding depends on several factors, which include length and composition of the stem region, loop size, temperature, and the salt concentration.^39–41^ The typical rate constants vary between 10^2^ and 10^6^ s^–1^ for hairpin folding.^39,41^ Finally, the IEEDA reaction between vinyl-fluorescein and tetrazine under a templated setting follow an observed first-order rate constant (*k*_1_) of the order 10^–4^ s^–1^.^29^ We first decided to investigate the kinetics of **HP3** activation by incubating the reporter gate with input DNA that contained between one and six nucleotides complementary to the single-stranded toehold domain of the blocking strand of the reporter duplex (Input_th1–6, Supporting Table 1). Moreover, the toehold domain was kept GC-rich, which favors strand displacement kinetics and would thus allow us to parse out the rate through the length of the toehold. Importantly, the reporter did not produce any fluorescence in the absence of an input strand, but an increase in fluorescence over time was observed upon incubation with input DNA, confirming that toehold-mediated strand displacement of the blocking strand by the input strand led to hairpin formation and templated fluorophore activation (Figure 2B). We further validated the successful reporter gate activation through mass identification of the activated hairpin product containing the unmasked fluorophore (MS Spectra—Supporting Information), formed upon completion of the IEDDA reaction. The rate of the reporter gate activation increased with an increasing length of the toehold domain on the input strand to a maximum toehold length of 5 nt. Notably, a 6 nt toehold did not lead to any further increase in fluorescence. We observed a *t*_1/2_ of 1.6 h for **HP3** (observed *k*_1_ = 1.19 × 10^–4^ s^–1^) when incubated with the 6 nt toehold input and a signal increase of 11.5-fold at 5 h. We observed similar trends for the rate of activation of **HP4** (*t*_1/2_ of 1.8 h and observed *k*_1_ = 1.07 × 10^–4^ s^–1^) for the 6 nt toehold input) (Figure 2B). Taken together, the similarity in kinetics between **HP3** and **HP4** and the direct relationship between toehold length and the rate of the fluorescence intensity increase support the conclusion that the displacement reaction of the inhibitor strand is the overall rate-limiting step.^34^ Given the modest performance enhancement of the methyltetrazine reporter relative to the pyridyl tetrazine, as well as evidence that suggest pyridyl tetrazines may possess less biological stability than methyl tetrazines,^42^ we chose to evaluate **HP3** in subsequent studies.

We additionally demonstrated that our hairpin reporter (**HP3**) resists false signal activation through nuclease degradation. Traditional quencher-based reporter gates use separate fluorophore- and quencher-labeled strands. Nuclease degradation separates these components, causing dequenching and false-positive signals (Supporting Figure S3A). In contrast, **HP3** employs a caged fluorophore activated only by a proximity-driven chemical reaction between the termini of its single-stranded hairpin structure. In this design, degradation releases inert fragments, preventing accidental activation. We compared **HP3** with an FQR control (matching **HP3**’s blocking strand sequence). Both reporters were fully degraded by DNase I (Supporting Figure S3B); however, **HP3** produced an output signal only when input strands triggered activation—no false-positive signal without input was observed (Supporting Figure S3C). In contrast, the classical FQR generated a false-positive signal in the absence of input due to fluorophore–quencher separation (Supporting Figure S3D). This confirms **HP3**’s stability against nuclease-induced false triggering, a significant advantage over conventional designs.

Following successful verification of the reporter gate functionality *in vitro*, we employed an imaging cytometry workflow to measure the activation in cells (Figure 3). Cells were transfected with independently encapsulated reporter and input oligonucleotides and incubated for 24 h to provide sufficient time for transfection and subsequent reporter gate activation. This method of independent encapsulation of input and reporter oligonucleotides does not result in cross-reactivity outside of the cell, as demonstrated by us and others.^30,43^ Cells were also transfected with the reporter gate alone as a control. Then, cells were fixed and stained for actin (rhodamine phalloidin) and nuclear DNA (Hoechst 33342). Using a 63× objective, a 6.5 μm *z*-stack was obtained to capture the entire cell depth. A maximum intensity projection was then generated and processed using a MATLAB plugin, FISH-quant.^44^ FISH-quant uses a series of filtering and denoising algorithms to evaluate the quality of fluorescent puncta in high-content imaging data sets.^45–49^ Similar cellular puncta were observed in other applications of intracellular strand displacement reporters and result from DNA circuits localized in endocytic vesicles.^30,43^ The overall workflow, as shown in Figure 3A, utilizes cell segmentation to provide the number of fluorescent spots detected per cell. Using this technique, we measured fluorescence activation in the presence and absence of input DNA and observed a 2-fold increase in the number of fluorescent spots detected per cell as a result of templated activation (Figure 3B). A considerable amount of variability in the number of spots detected per cell was observed. This variability can be attributed to differences between cells in the degree of colocalization within endocytic vesicles and in total cellular uptake of the input and reporter molecules, necessary for activation.^43^ Despite a reduced signal-to-background ratio in cells, compared to test tube experiments, the observed activation in the presence of input DNA versus the absence of input was highly statistically significant (*p* < 0.0001).

**Figure 3:**
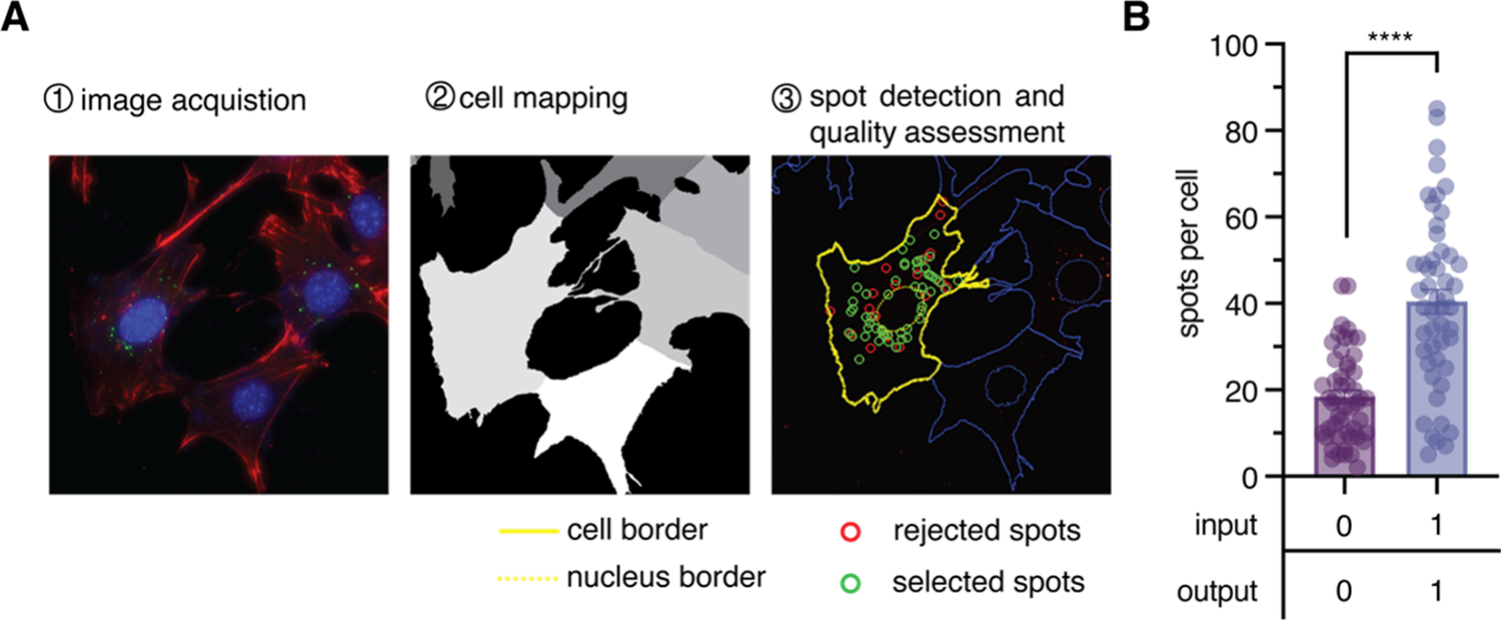
Activation of DNA hairpin reporter in cells. (A) Workflow for quantification of reporter activation in cells. Maximum intensity projections were generated from full cell *z*-stack images and used to detect the cell borders. Cell borders were overlaid on the filtered fluorescein fluorescence image to determine the number of spots per cell. Spot quality was accessed using the MATLAB script FISH-quant. (B) Quantification of cellular activation of the hairpin reporter. The mean number of spots per cell is shown ± s.e.m., *n* = 50. ****p < 0.0001; calculated from unpaired two-tailed Student’s *t* tests.

In addition to the detection of individual DNA or RNA species, DNA strand displacement devices are ideal for designing programmable networks of interacting DNA complexes. Using sequences with multiple layers of complementarity and distinct single-stranded toehold domains, it is possible to design the order of DNA strand displacement reactions. This enables the construction of complex DNA circuits, such as Boolean logic gates.^5,14,15,50–52^ To evaluate the performance of the hairpin-templated reaction reporter for integration into more complex DNA strand displacement cascades, we designed and tested an upstream OR gate that produces a TRUE output if one or both inputs are present (Figure 4). This gate consists of two double-stranded translator complexes, each of which releases a DNA strand complementary to the blocking strand of the hairpin reporter in the presence of one of two input DNAs. The OR circuit was first validated *in vitro* by monitoring the fluorescence intensity of four reaction mixtures composed of the caged reporter gate, translator gates 1 and 2, and each combination of inputs 1 and 2 (Figure 4C). Consistent with the expected OR gate truth table, the fluorescence intensity of the reaction mixtures containing input 1, input 2, or both input DNA strands showed a 5-fold increase in the signal relative to the reporter gate alone. The degree of activation is readily detectable, as illustrated by the statistically significant difference between the reporter gate alone and each input combination (*p* < 0.0001). This observation confirms the capability of integrating the hairpin reporter into more complex circuits. Having demonstrated the expected OR gate behavior in an *in vitro* assay, we then assessed the activity of the system in cells using the same workflow described above to quantify the amount of activation observed under each combination of input DNAs (Figure 4D,E). The results of this *in cellulo* analysis were consistent with the activation pattern observed *in vitro*. Cells cotransfected with either or both input DNA showed significantly more fluorescent spots per cell than those transfected with only the reporter and translator components, with an increase over background that showed high statistical significance (*p* < 0.0001) and permitted determination of successful OR gate operation in cells.

**Figure 4:**
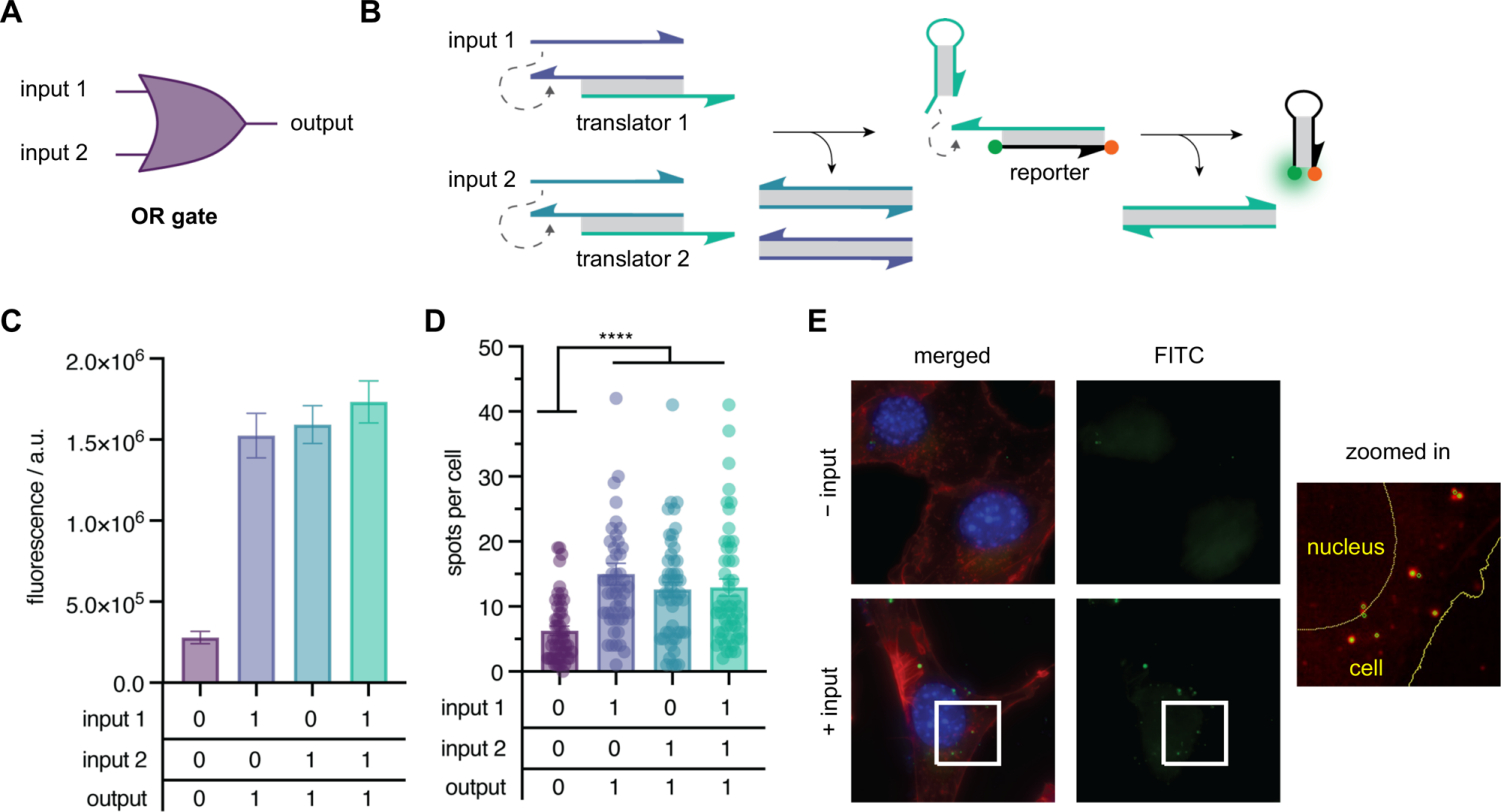
(A) OR gate symbol. (B) DNA strand displacement scheme. Input released from translator gate 1 or 2 hybridizes to the caged hairpin-based reporter and facilitates the templated IEDDA reaction between methyltetrazine (orange dot) and vinyl-ether-caged fluorescein (green dot) at opposing ends of the reporter. (C) Quantification of *in vitro* fluorescence activation of the OR circuit consisting of HPR (50 nM) and translators 1 and 2 (1.25×, 62.5 nM) in response to each input combination (2.5×, 250 nM). Mean fluorescence intensity is shown after 12 h of circuit incubation at 37 °C (mean ± s.d.; *n* = 3). (D) Activation of the OR circuit in cells. Mean fluorescent spots per cell are shown for a population of cells imaged under a 63× objective (mean ± s.e.m.; *n* = 50). ****p < 0.0001; calculated from multiple unpaired two-tailed Student’s *t* test. (E) Representative maximum intensity projections of cells.

We next sought to test the ability of our hairpin reporter gate to interface with a circuit designed to carry out AND logic (Figure 5A). An AND logic circuit will produce a TRUE output only if all inputs are present. The strand displacement scheme in Figure 5B depicts the series of strand displacement reactions in this circuit that implement the AND logic. First, input 1 hybridizes to the AND translator, forming a complete duplex (purple), exposing a toehold domain for input 2. Next, input 2 hybridizes to the exposed toehold on the translator and releases a strand complementary to the reporter gate. Lastly, the released input hybridizes to the toehold of the hairpin reporter and triggers activation. This strand displacement circuit was first verified in an *in vitro* fluorescence assay, which showed that in the presence of inputs 1 and 2, an over 2.5-fold increase in fluorescence was observed (*p* < 0.0001), reproducing the expected output with high statistical significance (Figure 5C). To further evaluate the performance of the circuit, cells were transfected with translator 1, translator 2, and reporter gates and each combination of input DNA. The quantified results from this experiment are shown in Figure 5D. As can be seen in the representative micrographs (Figure 5E), cells transfected with both input 1 and input 2 exhibited a greater mean number of fluorescent spots per cell than those transfected with only a single input or without input DNA, with a minimum fold change of approximately 2.5 over background (*p* < 0.0001). With this observation, we determined that the IEDDA reporter could be successfully implemented for two fundamental logic gates inside of cells.

**Figure 5:**
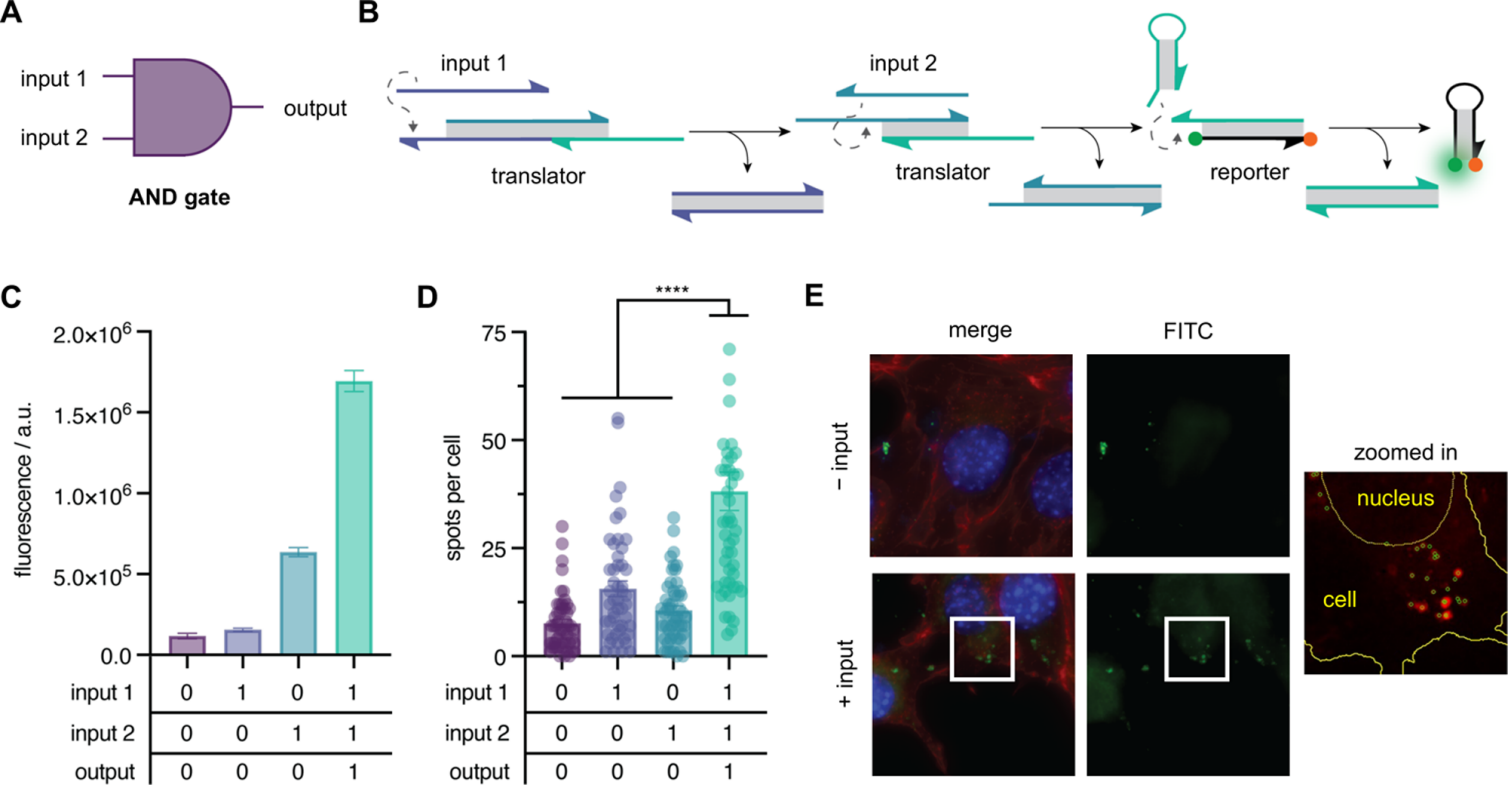
(A) AND gate symbol. (B) DNA strand displacement scheme. Input released from the AND translator gate hybridizes to the hairpin-based reporter and facilitates the templated IEDDA reaction between methyltetrazine (orange dot) and vinyl-ether-caged fluorescein (green dot) at opposing ends of the reporter. (C) Quantification of *in vitro* fluorescence activation of the AND circuit, consisting of HPR (50 nM) and the AND translator (1.25×, 62.5 nM), in response to each input combination (2.5×, 250 nM). Mean fluorescence intensity is shown after 12 h circuit incubation at 37 °C (mean ± s.d.; *n* = 3). (D) Activation of the AND circuit in cells. Mean fluorescent spots per cell are shown for a population of cells imaged under a 63× objective (mean ± s.e.m.; *n* = 50). ****p < 0.0001; calculated from multiple unpaired two-tailed Student’s *t* test. (E) Representative maximum intensity projections of cells transfected with reporter and translator gates with and without DNA inputs 1 and 2.

Similar to the OR circuit, the performance of the AND circuit did not reach the same level of overall activation compared to the *in vitro* data. This can again be attributed to the fraying or degradation of the reporter gate in cells. This contrasts with a multistrand reporter gate, which harbors the same reactive partners on two different DNA strands as opposed to our current hairpin design.^30^ Since both reactive groups are on the same strand, fraying of the blocking strand can lead to spurious folding of the hairpin resulting in gate activation. However, in our current design, the AND circuit (2.5-fold) performed better than the OR circuit (2-fold) in cells as depicted by higher activation over the background. This could be attributed to the degree of leakiness or nonspecific strand displacement, which is influenced by the nucleic acid sequence and circuit design. The overall background activation is likely to increase with increasing complexity of the strand displacement cascades and thus requires longer computation times. Furthermore, the hairpin reporter strand can be optimized in terms of the loop and stem length and composition. A combination of the above will lead to a generation of more stable and robust hairpin gates for *in cellulo* applications.

## Conclusions

In summary, an inverse molecular beacon reporter was developed that utilizes a hairpin-templated IEDDA reaction to effectively trigger the activation of a caged fluorophore in response to a specific input DNA sequence. The IEDDA reaction was implemented using a vinyl ether-caged fluorescein with both methyltetrazine and pyridinyl tetrazine reaction partners. We showed that reporter activation occurs as a result of toehold-mediated strand displacement reactions, for which the rate of activation can be tuned by changing the toehold length. In contrast to FRET-based inverse molecular beacons, which are rarely employed because the output consists of a reduction of fluorescence signal,^53^ our inverse, reaction-based molecular beacon reporter described here exhibits an up to 13-fold activation, due to small-molecule fluorophore activation as a result of templated chemical reaction. Additionally, the presence of both the reactive probes on the same DNA strands facilitates easier assembly of the reporter gate, bypassing the need to modify multiple DNA strands for a multistrand reporter gate. Furthermore, the ability of this reporter to integrate with DNA logic circuits and effectively transmit their output in the form of a fluorescent signal *in vitro* and *in cellulo* was demonstrated. The output of the inverse molecular beacon probe utilizes a templated chemical reaction, which can activate a functional small molecule in response to a specific nucleic acid input.^23,24,54,55^ While the inverse molecular beacon does not exhibit faster kinetics than a traditional fluorophore–quencher-based reporter, due to the rate-limiting chemical reaction, a significant improvement in activation over background was observed in the presence of nucleases. This trade-off between speed and signal-to-noise ratio may be acceptable in certain applications, for example, as a diagnostic/theranostic tool, where sensitivity and specificity are of greater importance than the reaction rate. The work presented here provides a framework for the design of next-generation reporters that consist of a single modified DNA oligonucleotide and undergo strand displacement reactions to promote DNA-templated activation.

## Experimental Section

### Logic Gate Preparation and Purification

DNA complexes were purified as previously reported.^50^ Briefly, gate duplexes were assembled at 20 μM in 100 μL of TE/Mg^2+^ buffer (Tris–HCl [10 mM; pH 8.0], EDTA [1 mM], and MgCl_2_ [12.5 mM]) and annealed by cooling the solution from 95 to 12 °C over 30 min in a thermal cycler (Bio-Rad, T100). Detailed descriptions for the assembly of individual gate complexes are included in the Supporting Information. Gates were then purified by a 16% native PAGE gel. The full-size duplex bands were identified using a hand-held UV lamp (4 W, Analytik Jena, UVL-21) via UV shadowing on a TLC plate, excised, cut into small pieces, and eluted overnight in 300 μL of TE/Mg^2+^ buffer. Gate concentrations were determined by UV absorption at 260 nm using a NanoDrop ND-1000 (Thermo Fisher Scientific) and calculated with the appropriate duplex extinction coefficient.

### Fluorescence Activation Measurement

Each reaction was prepared to a final volume of 50 μL in TE/Mg^2+^ buffer in a 384-well flat black plate (Greiner). Concentrated stock solutions of DNA gates used in reactions were prepared as described above and were generally between 2 and 10 μM, as determined by UV absorption at 260 nm. DNA inverse molecular beacon reporter (50 nM) and translator (62.5 nM, 1.25×) gates were mixed with the indicated combinations of input DNA (250 nM, 2.5×), and plates were sealed to limit evaporation. Fluorescence was measured on a Tecan M1000 Pro microplate reader (ex/em 492/522 nm) over 20 h with incubation at 37 °C in triplicate. Fluorescence intensity across experimental conditions was analyzed and plotted using Prism 9 (GraphPad). Fluorescence intensity versus time data was analyzed by fitting a nonlinear regression to determine the *t*_1/2_ of activation.

### Cell Culture

All cell culture experiments were performed in a sterile laminar flow hood. NIH 3T3 cells were maintained in Dulbecco’s Modified Eagle Medium (DMEM, Gibco, SH30003.03) supplemented with 10% (v/v) fetal bovine serum (Sigma-Aldrich, F0926) and 1% (v/v) penicillin/streptomycin (Corning, 30002CI) at 37 °C with 5% CO_2_. Cells in the culture were routinely tested for mycoplasma contamination.

### Fluorescence Imaging and Quantification

NIH3T3 cells were seeded into an 18-well chamber slide with a glass coverslip (ibidi, 81817, 15,000 cells/well) in 100 μL of DMEM. Following overnight incubation, media were replaced with 70 μL of DMEM (antibiotic-free). Cells were transfected with the indicated combination of DNA reporter (50 nM), translator (1.25×), and input (2.5×), all separately encapsulated in 10 μL of Opti-MEM transfection media using FuGENE HD transfection reagent (Promega, E2311, 3:1 μL reagent/ng DNA ratio). Following 24 h incubation, cells were washed twice with 50 μL of phosphate-buffered saline (PBS), fixed by immersion in 50 μL of 4% formaldehyde in PBS for 10 min at RT, and washed twice more with PBS. Fixed cells were stained in a 50 μL PBS solution containing 1× phalloidin rhodamine (Thermo Fisher, R415) and 300 nM DAPI (Invitrogen, D1306) F-actin and nuclei dyes for 15 min at RT and then washed twice and immersed in PBS. Whole-cell *z*-stack images (27 slices, 250 nm spacing) were obtained using SlideBook 6 imaging suite (3i) and a Zeiss Axio Observer Z1 with an LED light source (X-Cite LED Boost), a 63× oil immersion objective (Zeiss plan-apochromat), an sCMOS camera (Andor Zyla 4.2), and FITC (ex. 470/40, em. 525/50), TRITC (ex. 545/25, em. 605/70), and DAPI (ex. 395/25, em. 460/50) filter sets. Images were exported as TIFF files and processed using ImageJ (National Institute of Health). The number of fluorescent spots per cell in each image was quantified using FISH-quant.^44^

## Supplementary Material



## Notes

Detailed synthetic protocols, gate degradation experiment, sequence information, and supporting figures (PDF)
