# Supplementary material for: Inverted Molecular Beacons as Reaction-Based Hybridization Probes for Small-Molecule Activation by Nucleic Acid Inputs
Source: ACS Chem Biol. 2025 Jul 15;20(8):1990–8. doi: 10.1021/acschembio.5c00333 (PMC12362334; doi:10.1021/acschembio.5c00333)
Supplement: Supplementary file 1 [file cb5c00333_si_001.pdf]

Supporting Information

Inverted Molecular Beacons as Reaction-Based Hybridization Probes for Small Molecule Activation by Nucleic Acid Inputs

Cole Emanuelson, Anirban Bardhan, Nicholas Ankenbruck, Jessica Boette, and Alexander Deiters*

University of Pittsburgh, Department of Chemistry, Pittsburgh, PA 15260, USA

**To whom correspondence should be addressed: deiters@pitt.edu*

Experimental Methods and Materials 2

General Chemical Methods 2

Synthesis of Probes 2

Synthesis of Activatable Hairpin Reporter Gates (HP3 and HP4) 5

Supporting Figures 8

Supporting Table 1. 11

MS Spectra 12

References 14

Experimental Methods and Materials

General Chemical Methods

All reagents were purchased from commercial suppliers and used without further purification. All reactions for the synthesis of the chemical probes were performed in flame-dried glassware and stirred magnetically. Flash chromatography was performed by hand. NMR spectra were recorded on Bruker Ultrashield 300 MHz, 400 MHz, or 500 MHz spectrometers (Billerica, MA). Analytical LC-MS data was collected on a Shimadzu LCMS-2020 (Santa Clara, CA) and ionized via electrospray ionization (ESI, ±ve) method. High resolution mass spectrometry (HRMS) was performed by the University of Pittsburgh facilities using Thermo Scientific Q-Exactive Orbitrap (Waltham, MA) and ionized via electrospray ionization (ESI, ±ve) method.

Synthesis of Probes

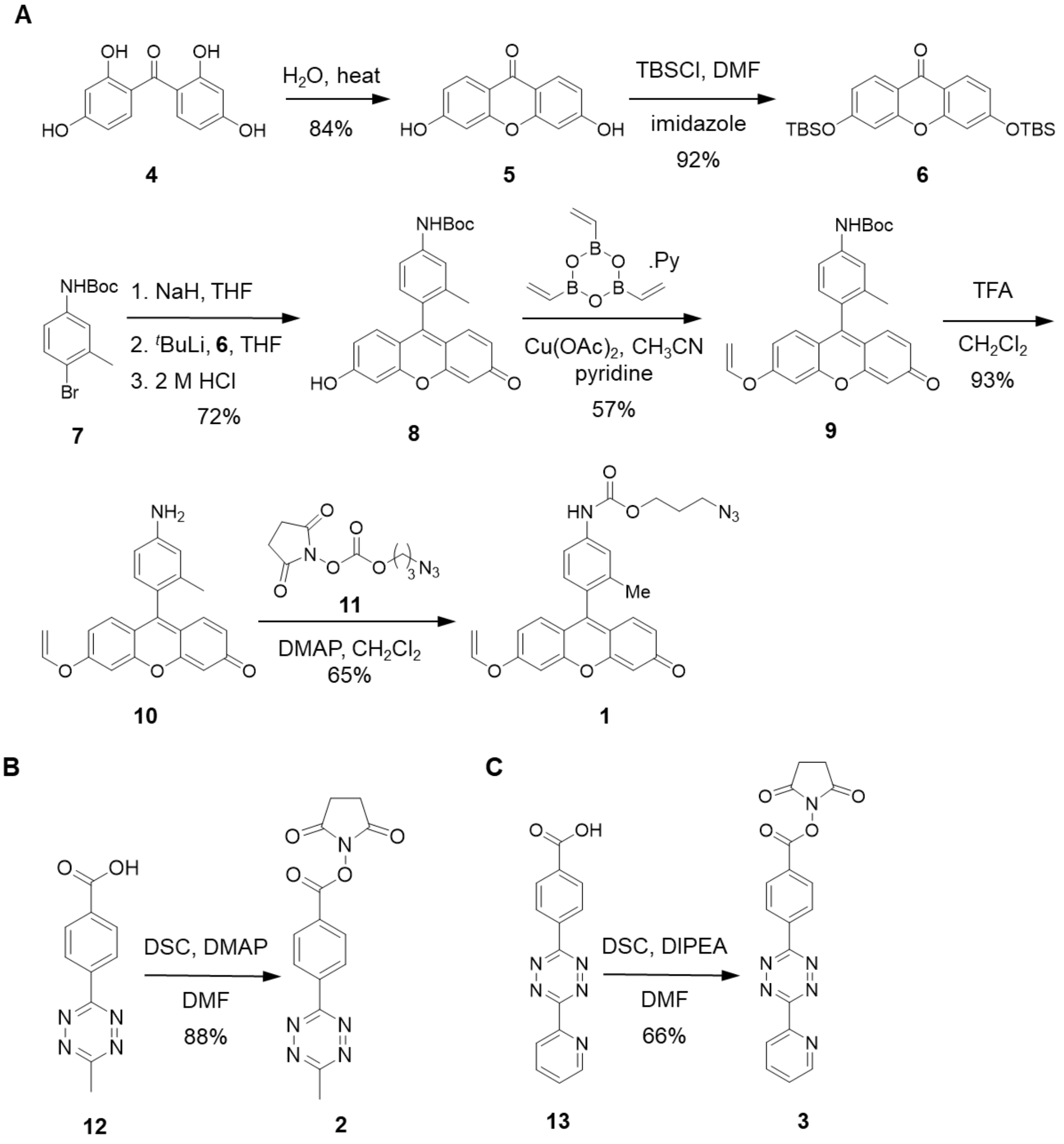

Supporting Scheme S1. Synthesis of probes used in this study. A) Synthesis of vinyl ether-caged fluorescein azide **1**, B) methyl tetrazine NHS ester **2**, and C) pyridinyl tetrazine NHS ester **3**.

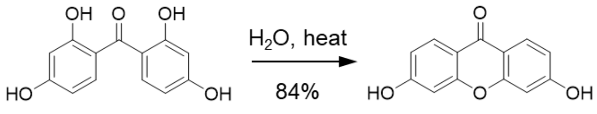

4 **5**

3,6-Dihydroxy-9H-xanthen-9-one (5). Xanthone **5** was synthesized from compound **1** following a previously established protocol and the analytical data matched reported results.[1]

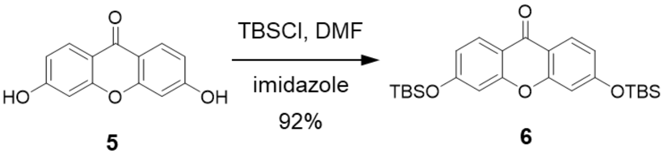

5 **6**

3,6-bis((*tert*-Butyldimethylsilyl)oxy)-9H-xanthen-9-one (6). Fluorescein xanthone **6** was synthesized from compound **5** following a previously established protocol and the analytical data matched reported results.[2]

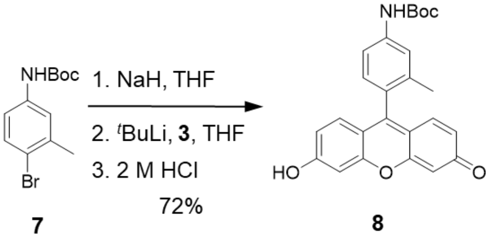

7 **8**

***tert*-Butyl (4-(6-hydroxy-3-oxo-9,9a-dihydro-3H-xanthen-9-yl)-3-methylphenyl)carbamate (8).** Fluorescein derivative **8** was synthesized from xanthone **7** following a previous report and the analytical data matched reported results.[3]

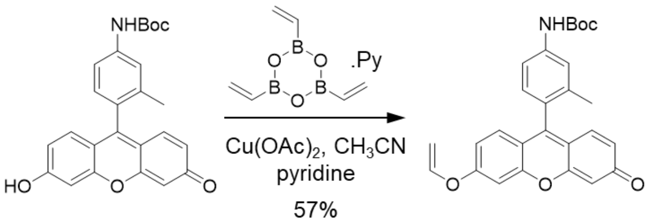

8 **9**

***tert*-Butyl (3-methyl-4-(3-oxo-6-(vinyloxy)-9,9a-dihydro-3H-xanthen-9-yl)phenyl)carbamate (9).** Copper (II) Acetate (26 mg, 0.14 mmol) was added to a flame-dried vial and dissolved in 2 mL of CH_3CN and was stirred at room temperature for 15 minutes. 2,4,6-Trivinylcyclotriboroxane-pyridine complex (27.4 mg, 0.11 mmol), compound **8** (60 mg, 0.14 mmol), and pyridine (0.12 mL, 14.3 mmol) were added to the reaction mixture. The mixture was then stirred at 40 °C for 36 h, followed by extraction with EtOAc (2 x 30 mL), washed with water (3 x 50 mL) and brine (50 mL) to remove salts and pyridine, before being dried over anhydrous Na_2SO_4 (1 g). The solvent was then removed in vacuo and the residue was purified by flash chromatography on silica gel, eluting with hexanes:EtOAc (1:2) to give the vinyl ether-caged fluorescein derivative **9** (36.2 mg, 57%) as an orange solid. The analytical data matched reported results.[3]

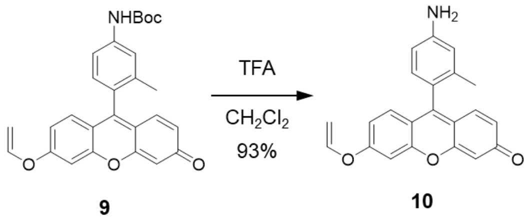

9-(4-Amino-2-methylphenyl)-6-(vinyloxy)-9,9a-dihydro-3H-xanthen-3-one (10). Vinyl ether-caged compound **9** (36.2 mg, 0.081 mmol) was dissolved in CH_2Cl_2 (3 mL) and cooled to 0 °C. Trifluoroacetic acid (0.33 mL; final concentration 10% v/v) was added to the solution and the mixture was stirred at 0 °C for 2 h. TLC showed complete spot to spot conversion of the starting material to the product. The reaction mixture was concentrated in vacuo and the crude product **10** (26.7 mg) was used directly for the next step without further purification. LCMS-ESI m/z calculated for $C_{22}H_{18}NO_3$ $[M+H]^+$: 344.12, observed 344.00.

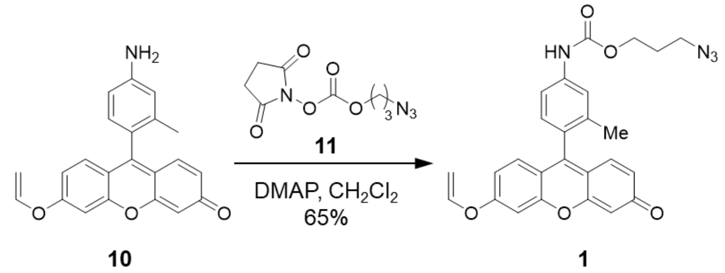

3-Azidopropyl (3-methyl-4-(3-oxo-6-(vinyloxy)-9,9a-dihydro-3*H*-xanthen-9-yl)phenyl) carbamate (1). To a chilled solution of compound **10** (20 mg, 0.057 mmol) in CH_2Cl_2 (1 mL) at 0 °C was added the propyl NHS ester **11** (21 mg, 0.087 mmol) and DMAP (14.2 mg, 0.114 mmol). The reaction mixture was left to warm up to room temperature overnight following which the solvent was evaporated *in vacuo*. The residue was then purified by flash chromatography on silica gel, eluting with CH_2Cl_2:MeOH (19:1) to furnish the vinyl ether-caged fluorescein azide **1** (18 mg, 68%) as a orangish-red solid. HRMS: m/z calculated for $C_{26}H_{22}N_4O_5$ $[M+H]^+$: 471.1663 Da; found [M+H]+: 471.1659 Da. ^{1}H-NMR spectrum of the compound is not included due to difficulty encountered in proper analysis owing to a poor signal to noise ratio. HPLC analysis was performed by dissolving a sample in acetonitrile (5% DMSO) at 500 uM concentration, followed by injection into a Shimadzu Prominence: Agilent Zorbax SB-C18 column (5 μM, 4.6 mm × 150 mm) and 30 min gradient of 5−95% acetonitrile in water. Note that the chromatogram was recorded after >30 months of storage at 20 °C in DMSO.

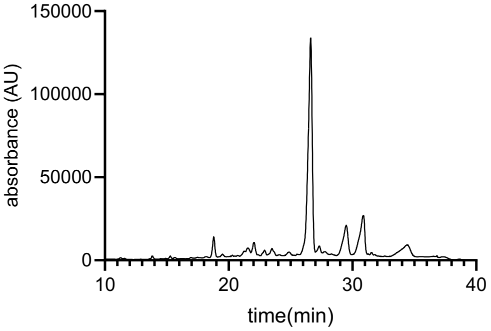

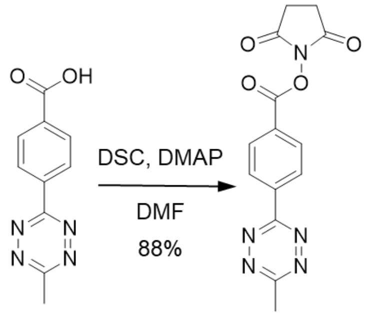

12 **2**

2,5-Dioxopyrrolidin-1-yl 4-(6-methyl-1,2,4,5-tetrazin-3-yl)benzoate (2). Compound **12** (15 mg, 0.07 mmol) was dissolved in DMF (2 mL) and to the solution was added *N,N'*-disuccinimidyl carbonate (36 mg, 0.14 mmol) and DMAP (21 mg, 0.18 mmol). The reaction mixture was left to stir overnight at room temperature following which the solvent was evaporated in vacuo. The residue was then purified by flash chromatography on silica gel, eluting with hexanes:EtOAc (1:4) to furnish the methyl tetrazine NHS ester **2** (19.2 mg, 88%). The analytical data matched previously reported results.[4]

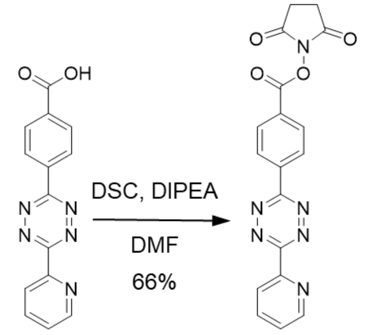

13 **3**

2,5-Dioxopyrrolidin-1-yl 4-(6-(pyridin-2-yl)-1,2,4,5-tetrazin-3-yl)benzoate (**3**). To a solution of the pyridinyl-phenyl tetrazine acid **13** (20 mg, 0.071 mmol) in DMF (1.5 mL) was added *N,N'*-disuccinimidyl carbonate (27.8 mg, 0.106 mmol), DIPEA (25 µL, 0.142 mmol). The reaction mixture was left to stir overnight at room temperature following which the solvent was evaporated in vacuo. The residue was then purified by flash chromatography on silica gel, eluting with CH_2Cl_2:MeOH (1:50) to furnish the diphenyl tetrazine NHS ester **3** (17.6 mg, 66%). ^1H NMR (400 MHz, $CDCl_3$) δ (ppm) = 8.96 (d, *J* = 2.3 Hz, 1H), 8.83 (d, *J* = 8.6 Hz, 2H), 8.63 (d, *J* = 7.9 Hz, 1H), 8.42 (d, *J* = 8.5 Hz, 2H), 8.18 (td, *J* = 2.1, 4.5 Hz, 1H), 7.78-7.73 (m, 1H), 2.94 (s, 4H).

Synthesis of Activatable Hairpin Reporter Gates (HP3 and HP4)

For the synthesis of the hairpin reporter gate **HP3**, the total volume of the reaction mixture is kept at 200 µL. To a 1.7 mL Eppendorf tube was added 100 µL of 200 µM of the 5'-alkyne-and 3'-amine modified hairpin strand **HP1** along with 10 µL of 20 mM vinyl ether-caged fluorescein azide **1** (1 mM final concentration) in DMSO. To the solution was added 44.5 µL of DMSO (final DMSO percentage is 30%), 10 µL of 2 M TEAA (final concentration of 0.1 M), 10 µL of Cu-TBTA solution (55% in DMSO, 0.5 mM final concentration), and 20.5 µL water. Finally, 5 µL of a 100 mM Na-ascorbate stock solution in water was added to bring the final volume of the mixture to 200 µL. The mixture was vortexed and incubated at room temperature for 2 h (but overnight incubations were performed as well). After incubation, 22 µL of a 3 M solution of sodium acetate in water was added to bring it to a final concentration of 0.3 M followed by addition of 800 µL of EtOH to the

reaction mixture. The mixture was cooled to –80 °C for 30 min to ensure complete precipitation of the hairpin strand **HP2**. The mixture was then centrifuged at 13,000 g at 4 °C followed by removal of the supernatant. Next, the pellet was washed with EtOH (2 x 400 µL) followed by 70% EtOH (2 x 400 µL), centrifuging each time for 10 min at 4 °C and removing the supernatant before proceeding with the next wash step. Following final centrifugation and removal of supernatant, the pellet was resuspended in 100 µL of water. The hairpin **HP2** was then analyzed/purified by HPLC using an ACE Excel 3 Oligo Beta Test C18 column (4.6 mm x 100 mm, 1.7 µM). Triethylammonium acetate buffer (0.1 M, pH = 7.0; refer to section 5.7.1 for TEAA buffer recipe) as used as solvent A and acetonitrile as solvent B. For analysis, 5 µL aliquot of the reaction mixture is diluted to 10 µL using milli-Q water and injected on the HPLC. The rest of the stock is purified using the HPLC in two batches (50 and 45 µL). The gradient utilized for analysis and purification was 5-33% solvent B over 25 min with a flow rate of 1 mL/min at 60 °C. The peak for **HP2** was monitored at both 260 nm and 480 nm and eluted at ~13.8 min. The purified **HP2** was concentrated using a cold speedvac and redissolved in 100 µL of milli-Q water.

Next, 50 µL of a 90 µM purified **HP2** stock in water was added to a 1.7 mL Eppendorf tube (final concentration of 30 µM) and to it was added 67.5 µL of the complementary blocking strand (final concentration of 45 µM; 1.5 eq,). To the solution was added 15 µL of 10X TE-Mg^{2+} buffer (pH 8.0) and 17.5 µL of water to bring the total volume up to 150 µL. The annealing step was then carried out on a thermal cycler using a temperature ramp from 25 °C to 95 °C over 2 min followed by a cool down from 95 °C to 15 °C over 25 min at a rate of 3.2 °C / min. An aliquot (10 µL) was analyzed on the HPLC using the same method but the column oven was kept at room temperature to verify successful annealing. Following verification, 100 µL of the mixture was pipetted into a new Eppendorf tube. To it was added 40 µL of 0.1 M $NaHCO_3$ buffer (pH = 8.5) followed by addition of 7.5 µL of 20 mM tetrazine (**2** or **3**; 50 eq.; final concentration of 0.75 mM) and 52.5 µL of DMSO (30% final DMSO concentration) to bring up the total volume to 200 µL. For the dipyridinyl tetrazine **3**, 30 µL of **3** was added along with 30 µL of DMSO to the mixture. The mixture was incubated at 4 °C overnight and 22 µL of 3 M sodium acetate (NaOAc) were added to the mixture (final concentration of 0.3 M) followed by addition of 800 µL of EtOH. The mixture was cooled to –80 °C for 30 min to ensure complete precipitation of the hairpin strand **HP3** or **HP4** oligonucleotide mixture. It was then centrifuged at 13,000 g at 4 °C followed by removal of the supernatant. Next, the pellet was washed with EtOH (2 x 400 µL) and 70% EtOH (2 x 400 µL), centrifuging each time for 10 min at 4 °C and removing the supernatant before proceeding with the next wash step. Following final centrifugation and removal of supernatant, the pellet was resuspended in 100 µL of 1X TE-Mg^{2+} buffer (pH = 8.0). The hairpin gate **HP3** was then analyze/purified on HPLC using the same method and gradient as described above, but the column oven temperature was kept at room temperature instead of 60 °C. For analysis, 10 µL of the resuspended solution was injected onto the HPLC while the rest was purified in two batches. Over the course of the HPLC runs, the final reporter gate **HP3** was monitored at 260 nm and eluted at ~14.8 min, right after the unreacted **HP2 duplex** (at ~13 min). The purified reporter gates **HP3** (at ~14.8 min) was concentrated down using a cold speedvac and redissolved in 100 µL by adding 1X TE-Mg^{2+} buffer. For the **HP4** analysis/purification, a gradient of 5-28% solvent B over 25 min with a flow rate of 1 mL/min at room temperature was used. The final reporter gate **HP4** (at ~18.9 min) was concentrated down in the same way and redissolved in 100 µL of 1X TE-Mg^{2+} buffer. The results for **HP3** and **HP4** assembly are depicted in **Supporting Figure S2**.

The peak at ~14.8 min was verified to be the desired reporter gate **HP3** was verified by collecting the peaks at ~13 min (unreacted **HP2 duplex**) and the peak at ~14.8 min (**HP3**) and subjecting them to a fluorescence activation assay. The **HP4** reporter gate was verified in a similar way.

.

Nuclease-mediated degradation experiment

Each reaction was prepared to 100 uL final volume in TE/Mg2+ buffer and analyzed in a 96-well flat black plate (Greiner). The classical reporter (FQR) was generated by annealing the two commercially available strands one with a fluorophore (FAM-5) and another complementary strand bearing a quencher (BHQ-1) (see **Supporting Table 1** for sequences). Annealing was performed by cooling a 1:1 solution of the two strands in TE/Mg2+ from 95 to 12 °C over 30 min in a thermal cycler (Bio-Rad, T100). The DNA reporter **HP3** was synthesized as described above and concentrated stock solutions of each reporter were prepared (10x, 500 nM), as determined by UV absorption at 260 nm. DNA reporters (50 nM) were mixed with DNase I (1 U/100 uL) and with or without input DNA (62.5 nM, 1.5x) and plates were sealed to limit evaporation. Fluorescence was measured on a Tecan M1000 Pro microplate reader (ex/em 492/522 nm) over 2 h with incubation at 37 °C in triplicate, and fluorescence intensity across experimental conditions was analyzed and plotted using Prism 9 (GraphPad).

Supporting Figures

A

Complex name	Equilibrium Complex Concentration (M)
HP1+HP1+HP1	1.65E-37
HPRblock+HPRblock+HPRblock	4.53E-35
HP1+HP1	2.33E-26
HPRblock+HPRblock	5.69E-24
HP1+HP1+HPRblock	7.64E-21
HP1+HPRblock+HPRblock	1.40E-17
HPRblock	4.39E-14
HP1	4.39E-14
HP1+HPRblock	5.00E-08

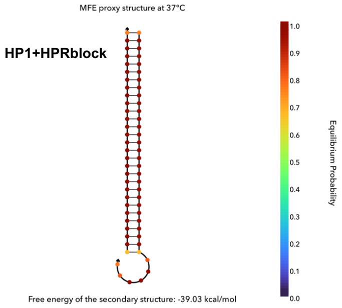

B

Complex name	Equilibrium Complex Concentration (M)
HPRblock+HPRblock+HPRblock	4.46E-64
HPRblock+HPRblock	2.61E-43
HP1+HPRblock+HPRblock	7.33E-31
HPRblock+HPRblock+input_th6	2.08E-30
Input_th6+input_th6+input_th6	4.37E-26
HPRblock	9.41E-24
HP1+input_th6+input_th6	1.41E-22
HP1+HP1+input_th6	3.79E-22
HP1+HP1+HP1	2.42E-19
HP1+HP1+HPRblock	2.12E-18
HPRblock+input_th6+input_th6	2.65E-18
HP1+input_th6	1.97E-17
Input_th6+input_th6	5.85E-17
HP1+input_th6+HPRblock	2.13E-16
HP1+HPRblock+input_th6	6.25E-16
HP1+HP1	3.01E-14
HP1+HPRblock	1.22E-11
input_th6	1.22E-11
HPRblock+input_th6	5.00E-08
HP	5.00E-08

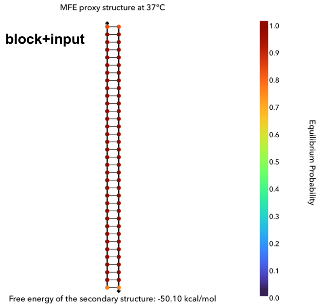

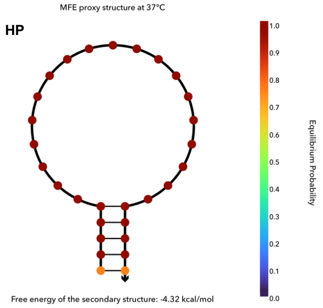

Supporting Figure S1. NUPACK analysis of the hairpin reporter gate. The predicted equilibrium concentrations of complexes expected to form in the 50 nM mixtures containing HP1 and HPRblock strands in A) absence and B) presence of input_th6 strand.

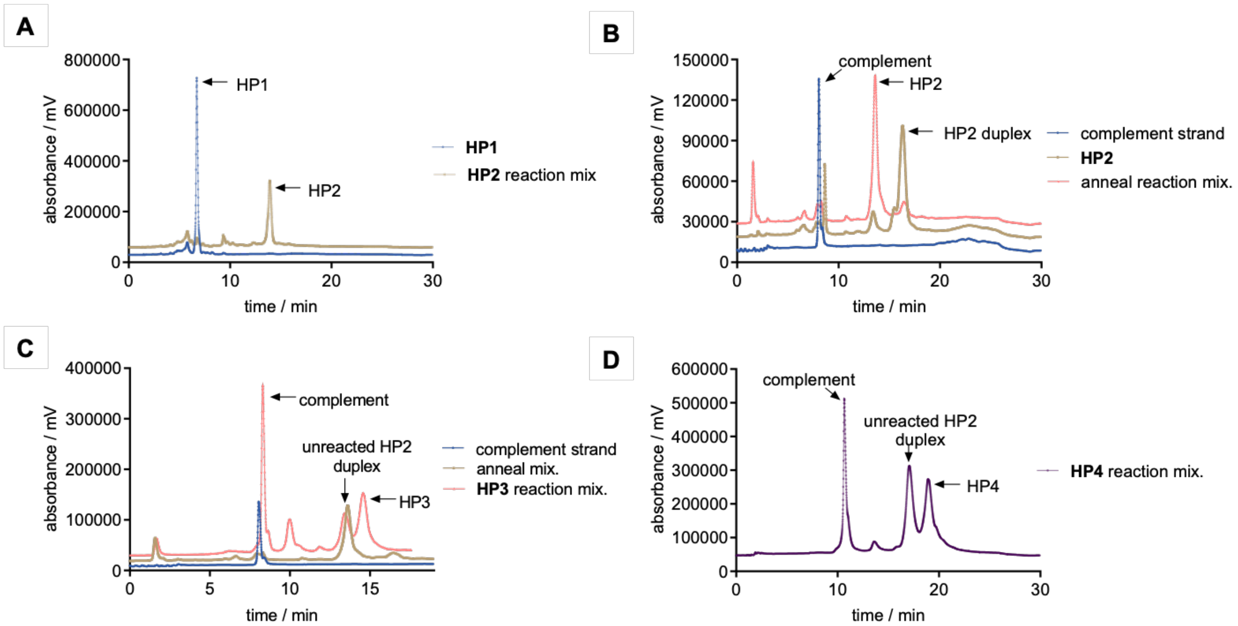

Supporting Figure S2. Assembly of the activatable reporter gate **HP3** and **HP4**. A) HPLC chromatogram of the conjugation of probe **1** with **HP1**. B) HPLC chromatogram of the annealing step of the complementary blocking strand with the **HP2** to generate the linearized HP2 duplex. C) HPLC chromatogram of the conjugation of the tetrazine probe **2** to the linearized **HP2** duplex for assembly of reporter gate **HP3**. D) HPLC chromatogram for the assembly of reporter gate **HP4** harboring a pyridinyl-substituted tetrazine upon reaction of **HP2** with **3**.

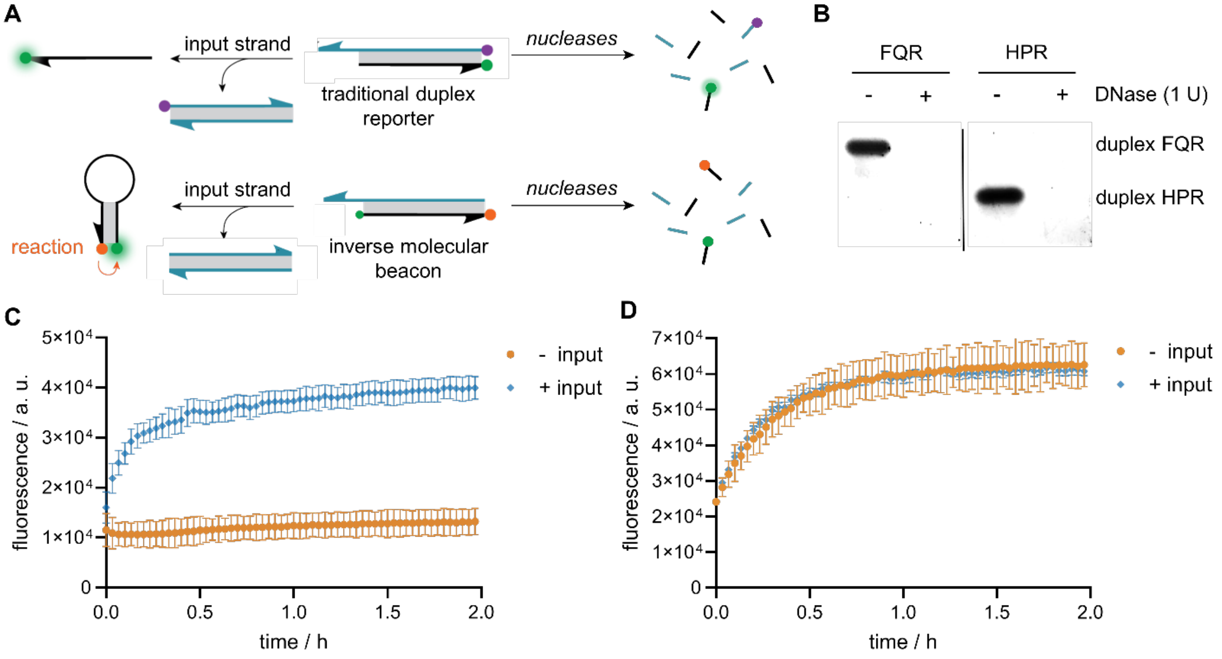

Supporting Figure S3. A) Impact of nuclease degradation on a traditional fluorophore-quencher reporter gate versus our inverse molecular beacon triggering a chemical reaction. B) Gel analysis of nuclease degradation of the inverse molecular beacon (HP3) and the fluorophore and quencher reporter (FQR). DNA duplexes (50 nM) were incubated with with DNase1 (1 U / 100 uL). C) **HP3** and D) FQR were tested their response to their respective input triggers in the presence of the endonuclease DNase **I**. Each reporter (50 nM) was incubated with or without the input release strand (62.5 nM) with the addition of DNase I (1 U / 100 uL). Fluorescence measurements (excitation = 492 nM, emission = 522 nM) were performed over 2 h. Experiments were conducted in triplicate and error bars represent standard deviation from the mean.

Supporting Table 1

Circuit(s)	Name	Sequence (5' to 3')
REP	Input_th6	CGGGCCGCGCGAAAAGAAGAAAAGGAAAAACGCGC
REP	Input_th5	GGGCCGCGCGAAAAGAAGAAAAGGAAAAACGCGC
REP	Input_th4	GGCCGCGCGAAAAGAAGAAAAGGAAAAACGCGC
REP	Input_th3	GCCGCGCGAAAAGAAGAAAAGGAAAAACGCGC
REP	Input_th2	CCGCGCGAAAAGAAGAAAAGGAAAAACGCGC
REP	Input_th1	CGCGCGAAAAGAAGAAAAGGAAAAACGCGC
OR	IN1	TCGTACCGTGAGTAATAATGCGCGGG
OR	TL1top	GGCCCGCGCATTATTACTCACGGTACGA
OR	TL1bottom	CGTGAGTAATAATGCGCGGGCCGCGCGAAAAGAAGAAAAGGAAAAACGCGC
OR	IN2	TCCTGTACTGAGCTGCCCCGAGCGGG
OR	TL2top	GGCCCGCTCGGGGCAGCTCAGTACAGGA
OR	TL2bottom	ACTGAGCTGCCCCGAGCGGGCCGCGCGAAAAGAAGAAAAGGAAAAACGCGC
AND	IN3	TAGCTTATCAGACTGATGTTGACGGG
AND	IN4	TGGAGTGTGACAATGGTGTTTGCGGG
AND	TL3th	TCAACATCAGTCTGATAAGCTATGGAGT
AND	TL3top	GGCCCGCAAACACCATTGTCACACTCCATAGCTTATCAGACTGA
AND	TL3bottom	GTGACAATGGTGTTTGCGGGCCGCGCGAAAAGAAGAAAAGGAAAAACGCGC
REP, OR, AND	HPRblock	GCGCGTTTTTCCTTTTCTTCTTTTCGCGCGGCCCG
REP, OR, AND	HP1	/5Hexynyl/GCGCGAAAAGAAGAAAAGGAAAAACGCGC/3AmMO/
REP, OR, AND	FAM-HP1	/56-FAM/CGGGCCGCGCGAAAAGAAGAAAAGGAAAAACGCGC
REP, OR, AND	BHQ-block	GCGCGTTTTTCCTTTTCTTCTTTTCGCGCGGCCCG/3-BHQ-1/

Mass Spectra

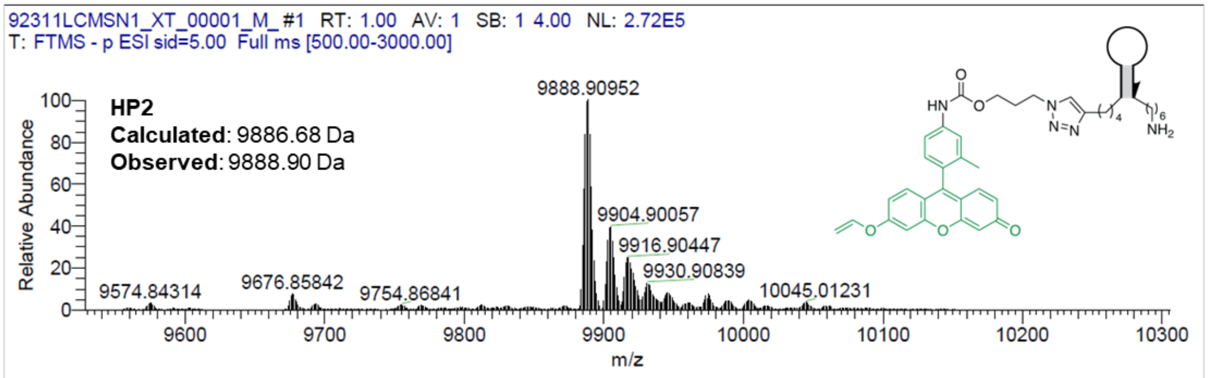

Mass analysis of HP3 reporter gate – ssDNA strand masses

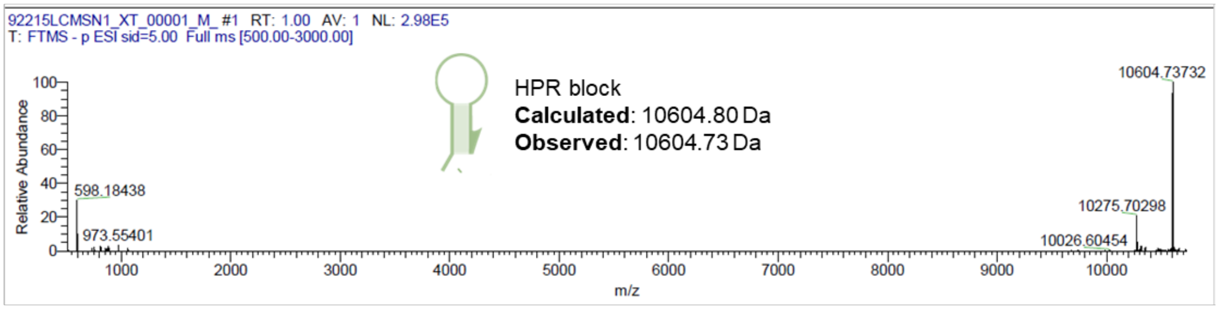

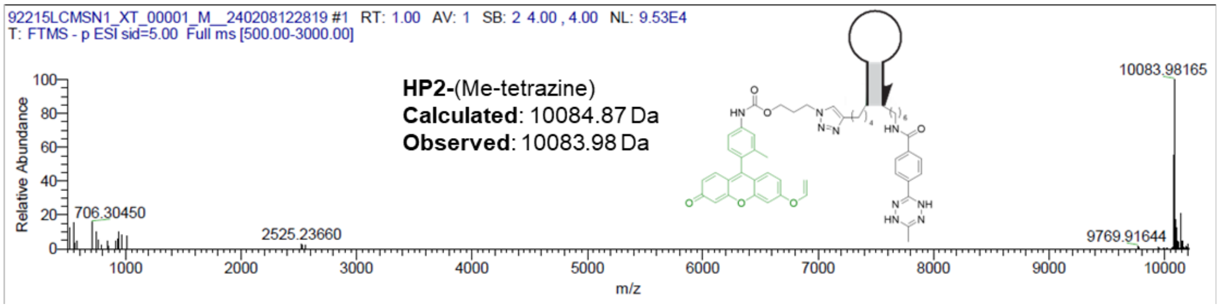

Mass analysis of HP4 reporter gate – ssDNA strand masses

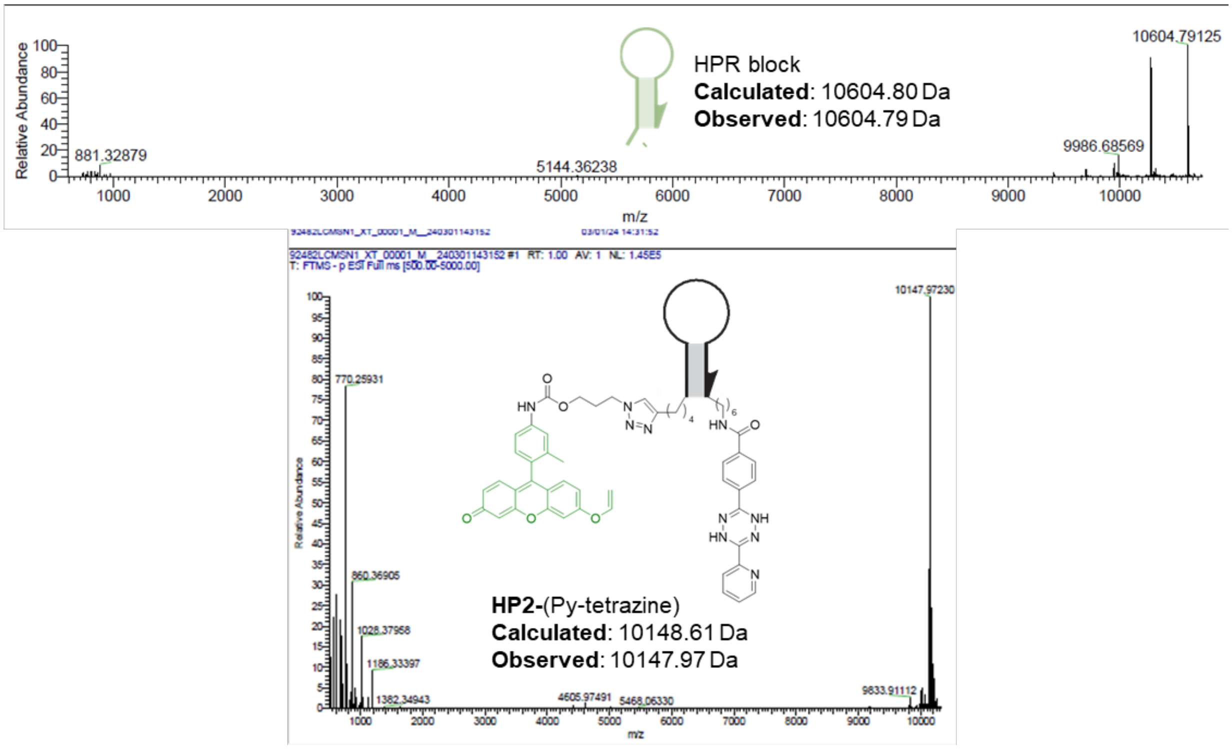

Mass analysis of HP4 reporter gate activation

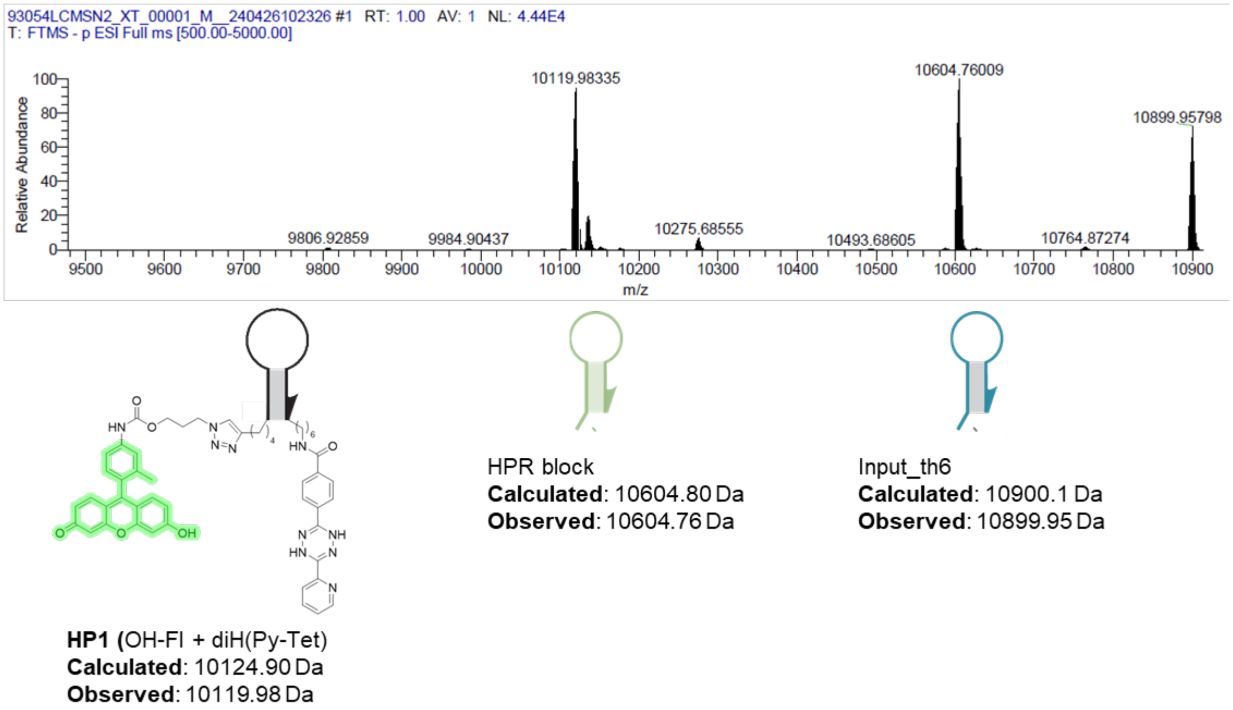

References

1. Piao, W.; Tsuda, S.; Tanaka, Y.; Maeda, S.; Liu, F.; Takahashi, S.; Kushida, Y.; Komatsu, T.; Ueno, T.; Terai, T.; Nakazawa, T.; Uchiyama, M.; Morokuma, K.; Nagano, T.; Hanaoka, K., Development of azo-based fluorescent probes to detect different levels of hypoxia. *Angew Chem Int Ed Engl* **2013,** *52* (49), 13028-32.
2. Shieh, P.; Hangauer, M. J.; Bertozzi, C. R., Fluorogenic azidofluoresceins for biological imaging. *J Am Chem Soc* **2012,** *134* (42), 17428-31.
3. Wu, H.; Alexander, S. C.; Jin, S.; Devaraj, N. K., A Bioorthogonal Near-Infrared Fluorogenic Probe for mRNA Detection. *J Am Chem Soc* **2016,** *138* (36), 11429-11432.
4. Tu, J.; Xu, M.; Parvez, S.; Peterson, R. T.; Franzini, R. M., Bioorthogonal Removal of 3-Isocyanopropyl Groups Enables the Controlled Release of Fluorophores and Drugs in Vivo. *J Am Chem Soc* **2018,** *140* (27), 8410-8414.
